# Resting-Associated Vocalization Emitted by Captive Asian House Shrews (*Suncus murinus*): Acoustic Structure and Variability in an Unusual Mammalian Vocalization

**DOI:** 10.1371/journal.pone.0111571

**Published:** 2014-11-12

**Authors:** Irena Schneiderová, Jan Zouhar

**Affiliations:** 1 Department of Game Management and Wildlife Biology, Faculty of Forestry and Wood Sciences, Czech University of Life Sciences, Prague, Czech Republic; 2 Department of Econometrics, Faculty of Informatics and Statistics, University of Economics, Prague, Czech Republic; Claremont Colleges, United States of America

## Abstract

Shrews have rich vocal repertoires that include vocalizations within the human audible frequency range and ultrasonic vocalizations. Here, we recorded and analyzed in detail the acoustic structure of a vocalization with unclear functional significance that was spontaneously produced by 15 adult, captive Asian house shrews (*Suncus murinus*) while they were lying motionless and resting in their nests. This vocalization was usually emitted repeatedly in a long series with regular intervals. It showed some structural variability; however, the shrews most frequently emitted a tonal, low-frequency vocalization with minimal frequency modulation and a low, non-vocal click that was clearly noticeable at its beginning. There was no effect of sex, but the acoustic structure of the analyzed vocalizations differed significantly between individual shrews. The encoded individuality was low, but it cannot be excluded that this individuality would allow discrimination of family members, i.e., a male and female with their young, collectively resting in a common nest. The question remains whether the Asian house shrews indeed perceive the presence of their mates, parents or young resting in a common nest via the resting-associated vocalization and whether they use it to discriminate among their family members. Additional studies are needed to explain the possible functional significance of resting-associated vocalizations emitted by captive Asian house shrews. Our study highlights that the acoustic communication of shrews is a relatively understudied topic, particularly considering that they are highly vocal mammals.

## Introduction

Mammalian vocal repertoires consist of a considerable amount of structurally variable vocalizations that are produced in different behavioral contexts. Some of these vocalizations, including territorial, advertisement, distress, contact, mating, echolocation, alarm, mobbing and food-associated vocalizations, have been studied extensively, and their meaning and diversity are currently fairly well understood [1]–[5]. However, the variability and functional significance of some vocalizations within mammalian vocal repertoires remain unclear, and many of these less well-studied vocalizations may be found among small mammals. For example, Pepper *et al.* [6] described a vocalization with unclear functional significance that was recorded only from reproductively active, urinating naked mole rats (*Heterocephalus glaber*).

Although the acoustic signals of shrews are often mentioned in relation to echolocation, the vocal repertoires of these insectivores include a considerable number of audible vocalizations [7]–[9]. The Asian house shrew (*Suncus murinus*) is a highly vocal insectivore species that emits a variety of vocalizations in different behavioral contexts, during the exploration of surroundings, courtship, aggressive or amicable intra-species encounters, and distressing situations [7], [9]. This is one of the most widespread and adaptable shrew species, and its impact on ecosystems and humans is non-negligible. This shrew species naturally occurs throughout the Indomalayan region and was introduced to coastal Arabia, eastern Africa and several islands in the Indian and Pacific Ocean, where it has caused a decline of the native species through competition and predation [10]–[12]. It was successfully domesticated as a laboratory animal in the United States and Japan and is occasionally kept as a pet; pet colonies were most likely established by animals imported from nature [13]–[15].

Recently, an unusual vocalization was observed and described in pet colonies of the Asian house shrew [9]. This tonal, low-frequency vocalization with a weakly modulated frequency can be clearly distinguished from other vocalizations occurring in the vocal repertoire of this species and is strictly tied to resting periods, when shrews are lying motionless in their nests. It is repeatedly emitted in a long series both by collectively resting shrews when they are maintaining physical contact with their siblings or mates in their nests and by singly resting shrews [9]. Audio S1 represents an example series of a resting-associated vocalization emitted by one resting male.

The production of such repetitive and, thus, relatively detectable vocalizations by resting Asian house shrews is remarkable because it has been previously demonstrated that vocalizations can attract predators or help predators find the nests of their prey [16]. Moreover, there is limited information regarding the existence and possible functional significance of a vocalization of this type in other shrews or mammals [17]–[19]. Thus, the first aim of our study was to provide a detailed description of the variability of this unusual and yet almost unexplored vocalization spontaneously produced by captive, resting Asian house shrews.

There is considerable intra-species variability regarding morphological, physiological and behavioral traits in the Asian house shrew [20]. Body size can also vary significantly according to locality, although males are usually significantly larger than females [20]. Louch *et al*. [21] reported an average body weight of 105.6 grams for males and 67.7 grams for females originating from Calcutta. Dryden [13] reported an average body weight of approximately 28 grams for two-month-old captive males originating from Guam and an average body weight of approximately 19 grams for females of the same age from the same locality. In mammals, differences in body size can be reflected by significant differences in vocalizations. Because the fundamental frequencies of vocalizations are generated by vocal folds and because larger vocal folds are capable of producing lower frequencies, lower fundamental frequencies are usually found in larger individuals within a species (see Matrosova *et al*. [22] for review). Moreover, lung capacity may influence the duration of produced sounds, with larger lungs being capable of producing longer vocalizations [23], [24]. Therefore, our second aim was to test whether there are differences in the acoustic structure of the resting-associated vocalizations produced by larger male and smaller female Asian house shrews.

Although it is a relatively widespread species, there is limited information regarding the social behavior and mating system of the Asian house shrew both in the wild and in captivity. Wild Asian house shrews are considered to be solitary and intolerant of one another; however, both males and females were observed to collect nesting material before the birth of juveniles, suggesting that this shrew may have a monogamous mating system [25], [26]. Domestic Asian house shrews from laboratory colonies are considered to be rather solitary, and individual housing is recommended in these colonies [14], [27], [28]. Conversely, pet breeders consider this shrew to be a fairly social species that can be successfully housed and bred in pairs and maintained in family groups. Under such conditions, collective resting of several individuals, including a male and female with their young, is usually manifested, and the resting-associated vocalization was first observed and described [9], [15].

Previous studies have showed that collective resting can be commonly found in some adult white-toothed shrews [19], [29], [30]. Such behavior may help reduce energy expenditure [30] or may result from a longer-term bond between males and females during the breeding period [29]. Naturally, young shrews also share a nest with their mother and siblings until they are weaned and become independent [13]. Olfactory and auditory cues may be helpful when maintaining contact between resting individuals sharing a common nest. Social cohesion, among other factors, may be mediated by signature vocalizations, i.e., vocalizations containing spectral and temporal acoustic features that allow conspecifics to discriminate between different individuals [31]. Such individually distinctive contact and cohesion vocalizations have been found in several mammalian species [32]–[34]. Thus, we also addressed the question of whether the resting-associated vocalization encodes information about the identity of the vocalizing Asian house shrews.

This study builds on a previous study describing an extensive vocal repertoire of the captive Asian house shrew [9] and focuses in more detail on a particular vocalization that is exclusively emitted by motionlessly lying and resting individuals. There is insufficient information regarding this type of vocalization in mammals. This study is one of the few that examines one specific vocalization emitted by any insectivore in detail and the only study that addresses the possibility that individuality is encoded in the vocalizations emitted by these small mammals.

## Methods

### Ethics statement

The Asian house shrew is listed as a species of least concern and is considered a fairly abundant and stable species with no direct conservation measures needed [12]. Neither keeping nor breeding this insectivore species requires any permission in the Czech Republic. Our research was conducted on animals from private breeding colonies. Therefore, private breeders (named in the Acknowledgements) were informed about our research aims and planned methods and subsequently they approved and permitted our research activities. The nature of our research did not require any manipulation or intervention concerning the welfare of the studied animals living in captivity.

### Study animals and housing

Resting-associated vocalizations were recorded from 15 adult Asian house shrews (eight males and seven females) living in two pet colonies located in Nymburk (one male and two females) and Prague (seven males and five females), Czech Republic. All of the shrews were born either in these colonies or in other colonies located in Germany. Shrews were considered adults when they were older than one month and could be housed separately from their mother and siblings. Data on the body weight of 11 individuals (five males and six females) from the Prague colony were known because these individuals were previously weighed using a digital scale (max 5000 g, ±1 g).

During this study, shrews were housed singly (one female together with a male) in VELAZ boxes (40×24×16 cm) (Nymburk) or IKEA plastic boxes (57×39×28 or 39×28×14 cm) (Prague). Each box was permanently labeled with the individuals' initials. Wood shavings were used for bedding, and the boxes were equipped with shelters (coconut shells or ceramic pots), cork bark, paper tubes, branches and running wheels. The shrews were fed *ad libitum* with various insects, meat and pellets, and water was always available.

### Vocalization recording

Recordings of the vocalizations were collected from shrews singly resting and spontaneously vocalizing either outside any shelter or hidden in a shelter using Marantz PMD-620 or 661 solid state recorders with internal microphones (16 bits, 44.1 kHz and 20–20,000 Hz) (D & M Professional, Kanagawa, Japan) from April 2010 to November 2011, using an *ad libitum* sampling method [35]. One female from Nymburk was recorded when resting outside a shelter together with a male but could be reliably identified as the vocalizing shrew because the exhalations accompanying the repeated vocalization were clearly recognizable due to chest movements. The distance between the resting shrews and the recording equipment ranged from 5 to 15 cm. One series of resting-associated vocalizations repeatedly produced during one resting period was recorded from most of the shrews. Only one male and one female provided two series recorded during two resting periods that were separated by a time span of two and 23 days, respectively. The duration of a single recorded series ranged from 27 seconds to 8 minutes and 58 seconds.

### Acoustic analysis

Acoustic analysis was conducted in Avisoft SASLab-Pro, version 5.1 (Specht, Berlin, Germany). The sampling frequency was reduced to 22.05 kHz in all of the recordings, which were subsequently visualized using spectrograms with the following settings: Hamming window, FFT length 512, frame size 50% and overlap 93.75%.

**Figure 1:**
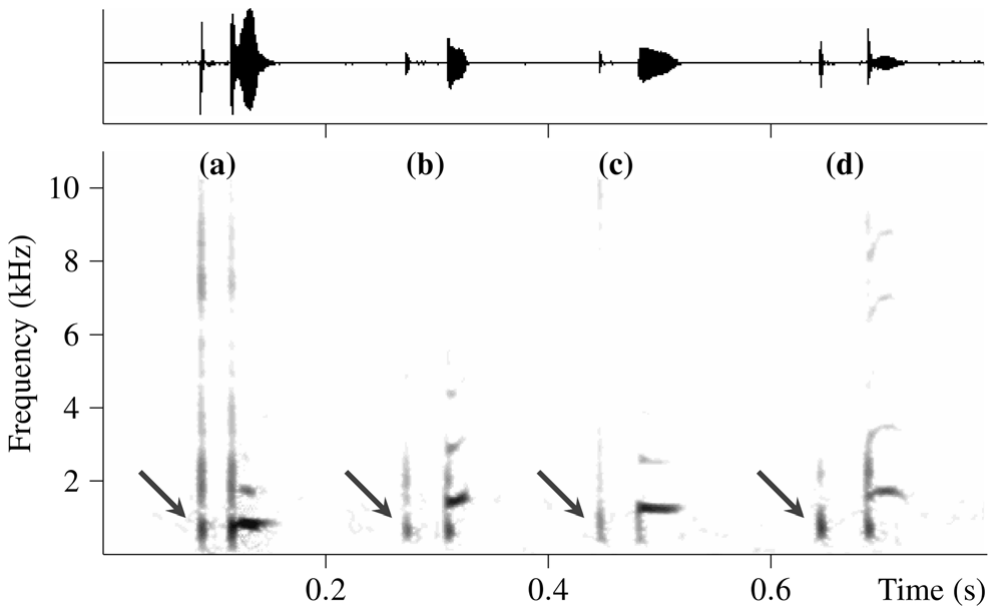
Representative resting-associated vocalizations emitted by captive Asian house shrews. Spectrogram (below) and waveform (above) of resting-associated vocalizations preceded by clearly visible non-vocal low clicks, with single low clicks (marked with arrows) simultaneously occurring in the recordings of one resting male (a) and three resting females (b), (c) and (d). Spectrogram settings: Hamming window, FFT length 512, frame size 50% and overlap 93.75%.

**Figure 2:**
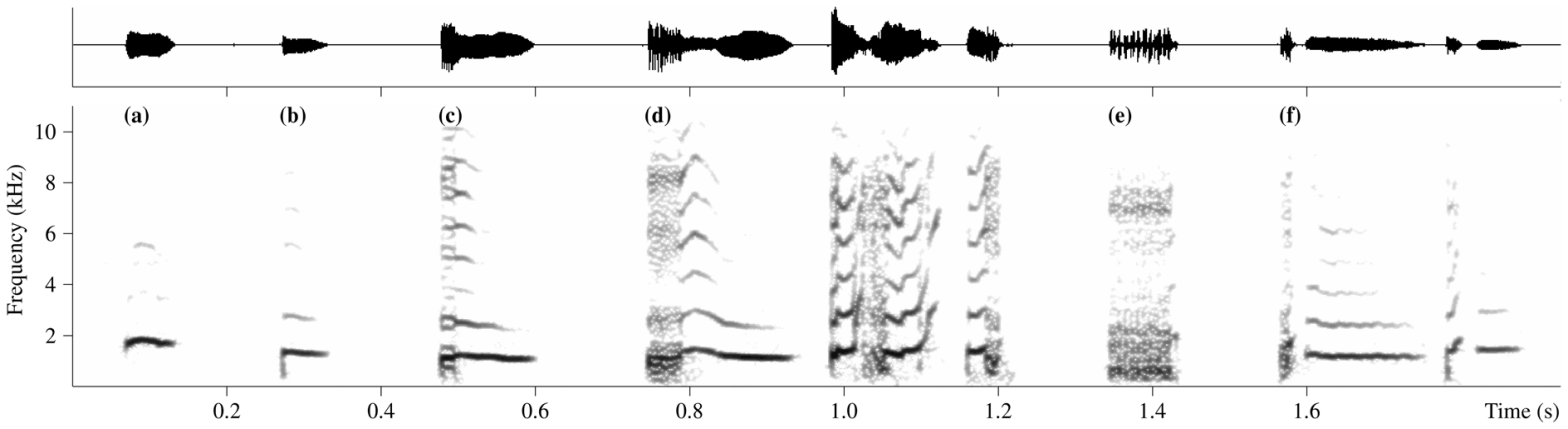
Structural variability of the resting-associated vocalizations produced by the captive Asian house shrew. Spectrogram (below) and waveform (above) of “tonal” (a), “tonal with low click” (b), “tonal with sub-harmonics” (c), “partially noisy” (d), “completely noisy” (e) and “separated in two parts” (f) resting-associated vocalizations. Spectrogram settings: Hamming window, FFT length 512, frame size 50% and overlap 93.75%.

Resting-associated vocalizations of a very low quality, e.g., those that (1) overlapped with background noise, (2) had a very low amplitude and (3) were of such a short duration that they were only with difficulty distinguishable from non-vocal low clicks also regularly produced by the resting shrews (Figure 1), were excluded from the analyses. A careful examination of the remaining vocalizations led us to classify them into one of six categories, as defined by their overall structural characteristics. Table 1 shows the six categories and their definitions, and Figure 2 shows representative spectrograms of these categories. In total, 1677 resting-associated vocalizations from 15 shrews (1157 from eight males and 520 from seven females) were categorized in this way. For each individual shrew, we expressed the occurrence of each resting-associated category as a percentage. Then, we averaged the percentages of all individuals per sex to obtain the average occurrence of each category in males and females.

**Table 1:** Summary and definitions of resting-associated vocalization categories found in the captive Asian house shrew.

Vocalization category	Definition
“Tonal”	tonal vocalization with the fundamental frequency (*f0*) and its harmonics
“Tonal with low click”	tonal vocalization with a low click clearly noticeable at the beginning
“Tonal with sub-harmonics”	tonal vocalization with sub-harmonics noticeable at the beginning
“Partially noisy”	partially tonal and partially noisy vocalization lacking *f0* and its harmonics
“Completely noisy”	vocalization completely lacking *f0* and its harmonics
“Separated in two parts”	vocalization separated in two parts where the first part can be either completely noisy or can start with a low click followed by rapidly increasing *f0* and the second part is tonal.

**Figure 3:**
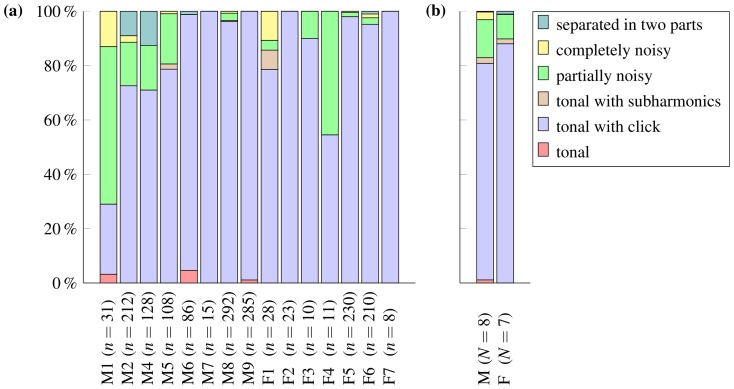
Occurrence of resting-associated vocalization categories in Asian house shrews. Occurrence of the six defined categories in each of the 15 studied Asian house shrew individuals (a) and in males and females (b). M =  male, F =  female, *N* =  number of individuals, *n* =  number of vocalizations.

A minimum of two and a maximum of 25 consecutive resting-associated vocalizations (251 vocalizations in total) of any category from one series were selected from each of the 13 individuals, eight males and five females, to measure the *interval*, i.e., the duration from the beginning of one vocalization to the beginning of the subsequent one. By definition, the consecutive vocalizations were not interspersed with other resting-associated vocalizations of low quality or even any low clicks, and these conditions were not met in two females. Therefore, *interval* could not be measured from these females. We also measured nine acoustic parameters for descriptive statistics and to perform univariate and multivariate statistical analyses to assess the variability both within and between sexes and individuals and to assess how many shrews could be potentially discriminated based on individuality, as encoded in the resting-associated vocalizations (Table 2). Because some categories (e.g., “tonal”, “tonal with sub-harmonics”, “completely noisy” and “separated in two parts”) were produced only rarely and their occurrence did not exceed 15% in any of the studied shrews (Figure 3), we excluded them from this acoustic analysis and related statistical analyses. Thus, a minimum of five and a maximum of 25 “tonal with low click” or “partially noisy” vocalizations (these categories reached more than 50% of all vocalizations produced by some individuals) of good quality and from one resting period were randomly selected from each of 15 studied shrews, eight males and seven females, to measure the acoustic parameters. In total, acoustic parameters were measured from 297 vocalizations, 178 from males and 119 from females.

**Table 2:** Summary and descriptions of acoustic parameters measured from randomly selected “tonal with low click” and “partially noisy” resting-associated vocalizations.

Acoustic parameter	Measuring procedure a	Definition and unit of measurement
*duration*	automatic	duration of the whole vocalization [ms]
*noisy percentage*	manual	proportion of duration that is noisy [% of vocalization's duration]
*peak location*	automatic	location of maximum amplitude within duration [% of vocalization's duration]
*peak f0*	automatic	frequency with the maximum amplitude of the vocalization [Hz]
*mean f0*	automatic	mean fundamental frequency (*f0*) of the vocalization [Hz]
*start f0*	manual	starting fundamental frequency (*f0*) of the vocalization [Hz]
*end f0*	manual	ending fundamental frequency (*f0*) of the vocalization [Hz]
*min f0*	manual	minimum fundamental frequency (*f0*) of the vocalization [Hz]
*max f0*	manual	maximum fundamental frequency (*f0*) of the vocalization [Hz]
*interval*	automatic	duration from the beginning of one vocalization to the beginning of a subsequent one [s]

a automatic  =  “Automatic parameter measurements tool” was used to extract parameters, manual  =  parameters were measured from the spectrogram window using “Free reticule cursor”.

### Statistical analysis

Statistical analyses were implemented in Stata 11 (StataCorp LP, College Station, TX, USA). Values of descriptive statistics of body weights and all measured acoustic parameters are reported as “*x* ± SD”. Differences in the group means of acoustic parameters between or within categories of resting-associated vocalizations were compared using *t*-tests, and we adjusted for the clustered nature of our dataset and treated individuals as primary sampling units (or clusters). In other words, we used cluster-robust variance estimates in these tests. Unless stated otherwise, all *p*-values are two-tailed, and tests are considered significant if *p*<0.01 and marginally significant if *p*<0.05.

In the following statistical analyses, data on the first nine acoustic parameters shown in Table 2 were used (all parameters except *interval*, which was, in general, measured from different vocalizations from the rest). Differences between sexes and individuals were tested using a series of nested two-way ANOVAs (one for each parameter), with individuality nested within sex. A parametric test could be used here because our data departed from normality (*p*<0.05) in only 6 of the 135 (4%) Kolmogorov-Smirnov tests performed.

To assess the number of individuals that could potentially be discriminated on the basis of the acoustic structure of their resting-associated vocalizations, we estimated the vocalizations' information capacity in bits per signal using Beecher's *H_S_* statistic [36]. We performed principal component analysis (PCA) and used all nine principal components (which can be extracted from our nine parameters) in the calculations. Because we did not aim to interpret the individual components, we did not apply any rotations to simplify the component structure. The information capacity of each component was calculated as 
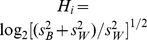
, where *H_i_* is the individualistic information content for the *i*-th component, 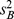 is the sum of squares “between” individuals, and 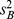 is the sum of squares “within” individuals. The total information capacity for a vocalization was calculated as 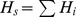 and was measured in bits; thus, the number of individuals that can potentially be distinguished using the encoded individuality can be calculated as 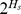.

As a complementary measure of individuals' vocalization distinctiveness, we used discriminant function analysis (DFA) to estimate the probability of correctly classifying our 15 shrews based on the acoustic structure of their resting-associated vocalizations. Due to our relatively small sample size, we opted for the conventional linear DFA (rather than either quadratic or logistic DFA). Prior classification probabilities were set as proportional to the number of vocalizations by the individual shrews. We used the leave-one-out method to reduce the upward bias in the estimation of the out-of-sample correct classification probability (CCP). Statistical significance of the estimated CCP was assessed through the permutation test suggested by Solow [37]. We ran DFA on 50,000 random permutations of the individuality indicator and calculated the CCP for each; we used the leave-one-out method again to make the results comparable to our baseline (i.e., non-permuted) CCP. The (one-tailed) *p* is estimated as the proportion of permutations that scored above the baseline CCP. Stata's “permute” function was central to our implementation.

## Results

### General acoustic structure of resting-associated vocalization

Analysis of the occurrence of the six structurally different categories of the resting-associated vocalization in the studied shrews showed that both sexes most frequently uttered the “tonal with low click” category. On average, this category included 80% of the resting-associated vocalizations produced by males and 88% of the resting-associated vocalizations produced by females. A clearly noticeable non-vocal low click could be observed at the beginning of each resting-associated vocalization that belonged to this category, and structurally identical low clicks could be found singly throughout the analyzed recordings (Figure 1). Therefore, we assume that this click occurring at the beginning of resting-associated vocalizations does not represent an acoustic artifact but is somehow produced by the resting shrews. For both sexes, the second-most frequently uttered vocalizations belonged to the “partially noisy” category, which represented, on average, 14% of the resting-associated vocalizations produced by males and 9% of the resting-associated vocalizations produced by females. Figure 3 shows the percentages of occurrence of all six categories of resting-associated vocalizations in each of the 15 studied shrews and the average occurrence in both sexes.

The resting shrews emitted the vocalizations almost continuously, with a relatively regular *interval* of an average duration of 1.4±0.9 s. The average *duration* of single vocalizations was 65±47 ms, with “partially noisy” vocalizations being, on average, about twice as long (115.5±92.3 ms) as “tonal with low click” vocalizations (57.5±29.0 ms), a result that was nevertheless only marginally significant (*t*_14_ = 2.70, *p* = 0.017). Naturally, the average “noisy percentage” was significantly higher (*t*_14_ = 3.77, *p* = 0.0021) in “partially noisy” resting-associated vocalizations (35.6±17.8%) than in “tonal with low click” vocalizations (21.0±11.7%). The average *mean f0*, most often coinciding with *peak f0* (92.3% of all vocalizations), was 1273±210 Hz. *Peak f0* also occasionally coincided with the first harmonic (7.7% of all vocalizations). Resting-associated vocalizations could be further characterized by minimal frequency modulation; the average *start f0* of 1251±197 Hz was almost the same as the average *end f0* of 1241±328 Hz (*t*_14_ = 0.24, *p* = 0.81). Descriptive statistics of all acoustic parameters measured from the “tonal with low click” and “partially noisy” resting-associated vocalizations are provided in Table 3.

**Table 3:** Descriptive statistics of acoustic parameters measured from randomly selected “tonal with low click” and “partially noisy” resting-associated vocalizations provided for males and females separately and for all studied individuals.

Acoustic parameter		Males		Females		All individuals	
		*N*/*n*	*x^−^*±SD	*N*/*n*	*x^−^*±SD	*N*/*n*	*x^−^*±SD
interval [s]		8/166	1.53±1.03	5/85	1.10±0.47	13/251	1.38±0.90
"Tonal with low click"	duration [ms]	8/149	68±40	7/110	43±21	15/259	58±29
	noisy percentage [%]	8/149	17±9	7/110	26±13	15/259	21±12
	peak location [%]	8/149	25±20	7/110	23±12	15/259	24±17
	peak *f0* [Hz]	8/149	1325±289	7/110	1492±391	15/259	1396±345
	mean *f0* [Hz]	8/149	1234±148	7/110	1377±233	15/259	1295±202
	start *f0* [Hz]	8/149	1205±137	7/110	1348±206	15/259	1266±189
	end *f0* [Hz]	8/149	1230±295	7/110	1350±329	15/259	1281±315
	min *f0* [Hz]	8/149	1004±152	7/110	1151±233	15/259	1066±203
	max *f0* [Hz]	8/149	1485±255	7/110	1585±290	15/259	1527±274
"Partially noisy"	duration [ms]	4/29	107±62	3/9	142±158	7/38	116±92
	noisy percentage [%]	4/29	33±18	3/9	45±12	7/38	36±18
	peak location [%]	4/29	24±21	3/9	25±25	7/38	24±22
	peak *f0* [Hz]	4/29	1053±177	3/9	1346±287	7/38	1122±240
	mean *f0* [Hz]	4/29	1068±191	3/9	1311±141	7/38	1126±207
	start *f0* [Hz]	4/29	1082±214	3/9	1378±242	7/38	1152±252
	end *f0* [Hz]	4/29	924±267	3/9	1124±318	7/38	971±289
	min *f0* [Hz]	4/29	760±245	3/9	999±301	7/38	817±275
	max *f0* [Hz]	4/29	1275±200	3/9	1576±268	7/38	1346±250

*N* =  number of individuals, *n* =  number of vocalizations.

### Sex and individual differences

**Figure 4:**
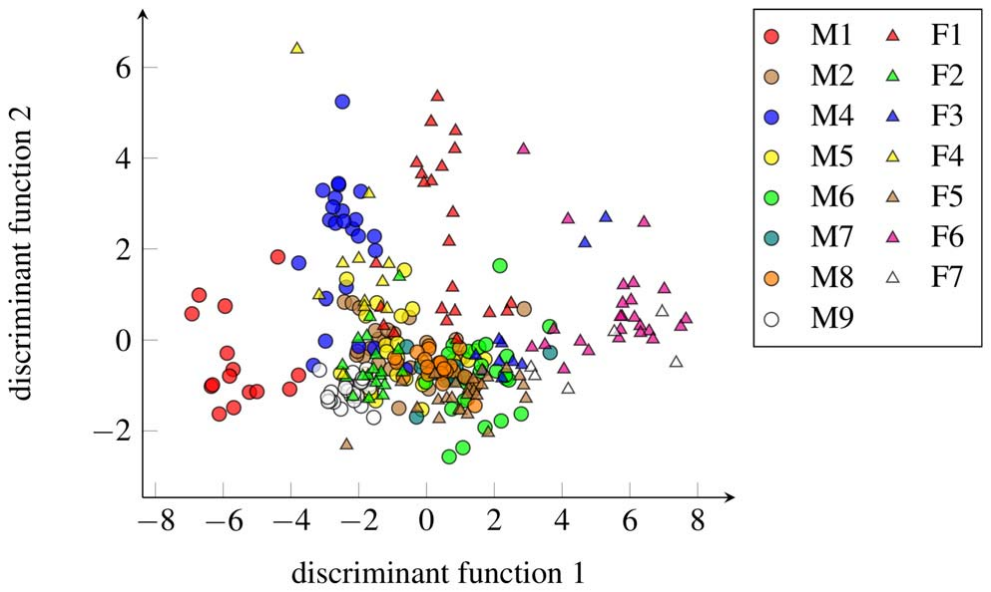
Scatterplot showing 297 resting-associated vocalizations recorded from 15 captive Asian house shrews separated by the first two discriminant functions. The first discriminant function was mainly correlated with frequency-related acoustic parameters (*peak f0* falling behind the rest), and the second discriminant function was correlated with the acoustic parameters *peak f0* and *noisy percentage*.

Males from the Prague colony were almost twice as heavy as females, with an average body weight of 66.2±13.4 grams and 39.2±5.4 grams, respectively. The results of nested two-way ANOVAs showed that there was no effect of sex, but there was a significant effect of individuality on all of the acoustic parameters (Table 4). Calculations of Beecher's information statistics showed that the total information capacity (*H_s_*) of the resting-associated vocalization equaled 3.0 bits, an amount of information that is sufficient to discriminate among eight individual shrews. DFA produced two discriminant functions with eigenvalues greater than 1, which together accounted for more than 79% of the total discriminating power (Figure 4). The first discriminant function (DF 1) was primarily correlated with frequency-related parameters (*peak f0* falling behind the rest), and the second discriminant function (DF 2) was correlated with *peak f0* and *noisy percentage*. The overall correct classification probability (CCP), calculated using the leave-one-out cross-validation procedure, was 60% (ranging from 8–95%) and was strongly significant (permutation test *p*<0.001). The estimated CCP corresponds with the estimated information capacity that allows discriminating among eight individual shrews. This finding implies that in a group of 15 individuals, the CCP should be slightly over 50%, which is consistent with our CCP estimate of 60%. See Beecher [36] for a discussion of the relationship between the CCP and *H*_S_. Figure 4 shows the separation of the resting-associated vocalizations recorded from all recorded individuals using the first two discriminant functions.

**Table 4:** Results of nested two-way ANOVA for effect of the factors sex and individuality on the acoustic parameters measured from resting-associated vocalizations of the studied Asian house shrews.

Acoustic parameter	Factor			
	Sex		Individuality	
	*F*_1,13_	*p*	*F*_13,282_	*p*
*duration*	1.57	0.23	8.08	0.00**
*noisy percentage*	2.70	0.12	10.79	0.00**
*peak location*	0.01	0.93	8.61	0.00**
*mean f0*	3.30	0.09	80.09	0.00**
*peak f0*	2.21	0.16	19.08	0.00**
*start f0*	3.95	0.07	69.41	0.00**
*end f0*	1.86	0.20	31.04	0.00**
*min f0*	3.14	0.10	52.13	0.00**
*max f0*	2.10	0.17	31.95	0.00**

Nested two-way ANOVA F-tests; ** *p*<0.001.

## Discussion

In this study, we recorded and analyzed resting-associated vocalizations that were spontaneously produced by 15 adult Asian house shrews living in captivity. This study is the first detailed description of an unusual vocalization with unclear adaptive significance emitted by any mammal exclusively during resting periods. Interestingly, Gould [7] did not identify the resting-associated vocalization in his study describing the vocal repertoire of captive Asian house shrews. Although differences in housing conditions or experimental design might have led to the initial identification of this vocalization in the Asian house shrew in a more recent study [9], it cannot be excluded that captive colonies of this species originating from different areas of its distribution manifest significant differences in their social and vocal behavior [7], [20].

There is an indication that structurally and contextually similar resting-associated vocalizations can be found in other white-toothed shrews and other mammalian taxa. Simeonovska-Nikolova [19] states that the bicolored white-toothed shrews (*Crocidura leucodon*) “produce sounds resembling low clucks when clustering and huddling up to the partner at group resting or before falling asleep”, and Fons [17] states that two pygmy white-toothed shrews (*Suncus etruscus*) can be often observed to “lie side by side in a common nest and emit plaintive calls whenever one of them moves”. Structurally and contextually similar vocalization has also been described in the Senegal bush baby (*Galago senegalensis*) by Zimmermann [18], who concluded that this vocalization is associated with the REM-sleeping phase and is most likely correlated with dreaming. Although the rare and accidental vocalizations that can sometimes be heard from resting and sleeping mammals are most likely associated with dreaming or slight disturbances during their sleep [8], [18], the nature of the vocalization emitted by resting Asian house shrews, particularly its frequent emission in long series with regular intervals, suggests that it might have some functional significance for this species. Vocalizations analyzed in this study were primarily recorded from singly resting shrews to provide reliable data collection, but preliminary observations showed that these resting vocalizations are also frequently emitted by pairs or siblings sharing a common nest [9], suggesting that they might be important for social cohesion and maintaining contact during resting periods. The presence of non-linear phenomena in the vocalizations emitted by resting Asian house shrews, particularly the presence of “partially noisy” vocalizations, suggests that shrews may also use these vocalizations to indicate their motivational state, e.g., drawing attention to their discomfort, when sharing their nest with other family members. Although it cannot be excluded that “partially noisy” vocalizations represent a non-adaptive by-product resulting from the activity of the vocal apparatus, their unpredictable nature makes them difficult to ignore [38].

Despite their obvious differences in body size, the resting-associated vocalizations of male and female Asian house shrews did not differ in principle. Rather, our results suggest that this vocalization manifests structural variability that could allow for the short-term discrimination of several shrews. However, coincident with the simple acoustic structure of this vocalization, the encoded individual variability was low compared with other types of vocalizations produced by socially or colonially living mammals [39]–[41]. Nonetheless, it is possible that even this low individual variability, which allows for the short-term discrimination of approximately eight individuals, is sufficient for the shrews to discriminate among members of a small family, i.e., male and female with several young, collectively resting in a common nest and uttering the resting-associated vocalization. Litter size varies from one to eight juveniles in the Asian house shrew, depending on their locality of origin [20], and young Asian house shrews are capable of producing the resting-associated vocalization after their eyes are opened, at approximately ten days of age [9], [20]. Juveniles still occupy a common nest at this age, at least until the age of approximately 20 days, when caravanning, a typical behavior displayed by young white-toothed shrews and their mothers, starts to disappear [20], [42]. However, the question remains whether the resting Asian house shrews indeed perceive the presence of their mates, parents or young in a common nest via the resting-associated vocalization and are capable of discriminating these individuals.

Given that the resting-associated vocalization in the Asian house shrew is quite noticeable, it is interesting that apart from two more white-toothed shrews [17], [19] and the Senegal bush baby [18], there is not much evidence in the literature [see, e.g., [6],[43],[44],[45]] suggesting that this vocalizationis a common part of the vocal repertoires of other small mammals (but see Credner *et al.* [46] for a description of tooth grinding recorded in adult resting and sleeping mole rats *Cryptomys* sp.). Is this type of vocalization indeed a rare phenomenon occurring in some captive colonies or specific taxa only, or has it remained undetected in some species? Many studies focusing on the vocal repertoires of mammals have been conducted in natural and semi-natural habitats, where animals can hide from researchers during resting periods [47], [48]. Considering captive individuals, the experimental design of some previous studies conducted in laboratories most likely did not allow for the detection and recording of spontaneously produced resting-associated vocalizations [7], [49].

Unfortunately, the terms and nature of our study did not allow us to answer more specific questions regarding the resting-associated vocalizations emitted by Asian house shrews, and additional studies are needed to explain its functional significance. Further studies should include behavioral and playback experiments, which would more adequately test the possible communicative significance of resting-associated vocalization in this species. Regardless, our study describing a new vocalization with unclear functional significance in the Asian house shrew demonstrates that acoustic communication in shrews represents a relatively understudied topic, particularly because they are highly vocal mammals. Descriptive studies concerning inter-species or intra-species variability [50]–[53] and experimental studies investigating the informational content and functional significance of shrews' acoustic signals [54] are still scarce in comparison with the numerous studies devoted to vocalizations in other mammalian taxa.

## Supporting Information

Table S1 **Data on body weight in 11 Asian house shrews and data on the type of resting-associated vocalization and the acoustic parameters measured from these vocalizations in 15 Asian house shrews.**
(XLS)

Audio S1 **A short series of resting-associated vocalizations spontaneously emitted by one motionlessly lying and resting male Asian house shrew.**
(WAV)
